# Reduced density of glutamine synthetase immunoreactive astrocytes in different cortical areas in major depression but not in bipolar I disorder

**DOI:** 10.3389/fncel.2015.00273

**Published:** 2015-08-10

**Authors:** Hans-Gert Bernstein, Gabriela Meyer-Lotz, Henrik Dobrowolny, Jana Bannier, Johann Steiner, Martin Walter, Bernhard Bogerts

**Affiliations:** ^1^Department of Psychiatry, University of MagdeburgMagdeburg, Germany; ^2^Clinical Affective Neuroimaging Laboratory, University of MagdeburgMagdeburg, Germany

**Keywords:** major depression, bipolar disorder, cortex, glutamine synthetase, astroglia, oligodendroglia, immunocytochemistry

## Abstract

There is increasing evidence for disturbances within the glutamate system in patients with affective disorders, which involve disruptions of the glutamate–glutamine-cycle. The mainly astroglia-located enzyme glutamine synthetase (GS) catalyzes the ATP-dependent condensation of ammonia and glutamate to form glutamine, thus playing a central role in glutamate and glutamine homoeostasis. However, GS is also expressed in numerous oligodendrocytes (OLs), another class of glial cells implicated in mood disorder pathology. To learn more about the role of glia-associated GS in mental illnesses, we decided to find out if numerical densities of glial cells immunostained for the enzyme protein differ between subjects with major depressive disorder, bipolar disorder (BD), and psychically healthy control cases. Counting of GS expressing astrocytes (ACs) and OLs in eight cortical and two subcortical brain regions of subjects with mood disorder (*N* = 14), BD (*N* = 15), and controls (*N* = 16) revealed that in major depression the densities of ACs were significantly reduced in some cortical but not subcortical gray matter areas, whereas no changes were found for OLs. In BD no alterations of GS-immunoreactive glia were found. From our findings we conclude that (1) GS expressing ACs are prominently involved in glutamate-related disturbances in major depression, but not in BD and (2) GS expressing OLs, though being present in significant numbers in prefrontal cortical areas, play a minor (if any) role in mood disorder pathology. The latter assumption is supported by findings of others showing that – at least in the mouse brain cortex – GS immunoreactive oligodendroglial cells are unable to contribute to the glutamate–glutamine-cycle due to the complete lack of amino acid transporters (Takasaki et al., 2010).

## Introduction

Initially, search for the possible cellular substrate of mood disorder pathology had focused on neurons (Rajkowska, 2000, 2002; Cotter et al., 2001a,b, 2002; Manji et al., 2001). However, during the last years a wave of information appeared to suggest that glial cells prominently, and in many ways, contribute to brain structural and functional changes in mood disorders. In numerous histological postmortem investigations both significantly reduced (Öngür et al., 1998; Rajkowska, 2000; Cotter et al., 2001a,b; Hamidi et al., 2004; Uranova et al., 2004; Rajkowska and Miguel-Hidalgo, 2007; Altshuler et al., 2010; Gos et al., 2013) and increased (Davis et al., 2002; Mosebach et al., 2013; Malchow et al., 2014) glial cell numbers and numerical densities have been observed in prefrontal cortex areas and limbic regions. In addition, characteristic changes in gene expression patterns and metabolic pathways of glial cells have been found in affective disorders (reviewed in Barley et al., 2009; Steiner et al., 2012; Mosebach et al., 2013; Duncan et al., 2014; Schroeter et al., 2014; Bernstein et al., 2015). Of note, each of the three glial cell classes (i.e., ACs, OLs, and microglia) appears to confer its unique contribution to the pathophysiology of affective disorders. Moreover, their impact may be different in MDD (Verkhratsky et al., 2014) and in BD, (Savitz et al., 2014; Dong and Zhen, 2015). In this context, much attention has been paid to the possible role of ACs in MDD and BD pathology. Being the most prevalent cell type in the human brain, ACs serve a wide range of different functions in the CNS. They play important roles in synaptic transmission, control of neuronal metabolism, sensing of brain microenvironment, maintenance of blood-brain barrier integrity, brain defense and inflammatory processes (Sofroniew and Vinters, 2010; Teschemacher et al., 2015). Since synaptic, metabolic and inflammatory dysregulation are reported in MDD and BD (Harrison, 2002; Si et al., 2004; Kato et al., 2013; Duman, 2014; Maletic and Raison, 2014; and others), AC abnormalities may be implicated in these disorders. ACs exert influence on cerebral information processing mainly in two ways: (1) they release, by exocytosis, gliotransmitters (glutamate, D-serine, GABA and ATP, Sahlender et al., 2014), which facilitate the communication between neurons and neuron-glia crosstalk, and (2) they remove glutamate from extracellular space and provide glutamatergic and GABAergic neurons with glutamine, which they synthesize from glutamate, ammonia and ATP (Anlauf and Derouiche, 2013). Theoretically, impaired release of gliotransmitters (Van Horn et al., 2013) and/or compromised glutamate uptake and recycling by ACs might significantly contribute to anomalies of glutamatergic (and, most probably, GABAergic) neurotransmission reported in MDD and BD (for reviews, see Walter et al., 2009; Brennan et al., 2010; Gigante et al., 2012; Salvadore et al., 2012; Bernstein et al., 2013; Dou et al., 2013; Duman, 2014; Arnone et al., 2015; Pehrson and Sanchez, 2015). However, while knowledge about the possible impact of gliotransmitters on mood disorder pathology is still fragmentary (Etiévant et al., 2013), there is some good evidence in favor of impaired AC-related glutamate-glutamine cycling as an important contributing factor in affective disorders (reviewed in detail by Bernstein et al., 2013 and Rajkowska and Stockmeier, 2013). Due to its central position in the glutamate–glutamine–GABA cycle, the glutamine-synthesizing enzyme GS (aka glutamate-ammonia ligase, EC 6.3.1.2) came early into focus of research, and a number of papers have meanwhile been published about alterations in GS in MDD and BD. In subjects with MDD a majority of studies show a decrease of cerebral GS. The expression of GS mRNA was reported to be down-regulated in the prefrontal cortex, the premotor cortex, and the amygdala of depressed suicide victims but not in suicide completers without depression (Choudary et al., 2005; Sequeira et al., 2009), whereas GS protein was found to be reduced in the anterior cingulate cortex and the orbitofrontal cortex (Choudary et al., 2005; Beasley et al., 2006; Miguel-Hidalgo et al., 2010; Rajkowska and Stockmeier, 2013). However, no alterations in cortical GS of MDD subjects were found by others (Toro et al., 2006). The implication of GS for BD is less well explored, and the sparse data are inconsistent. GS expression was reported unchanged (Toro et al., 2006) or decreased (Choudary et al., 2005) in the prefrontal cortex of bipolar subjects. Remarkably, all but one (Toro et al., 2006) studies on GS expression in mood disorders used biochemical techniques. Thus, little attention has yet been paid to the type of GS-expressing cells, although there is evidence that GS can also be detected in extra-astroglial localizations [i.e., in numerous OLs and even in some neurons (Takasaki et al., 2010; Iwata et al., 2013; Bernstein et al., 2014 and others)]. Thus, theoretically, disease-related alterations of GS expression might involve non-astroglial cells, too. We, therefore, counted GS expressing glial cells in ten different cortical and subcortical brain areas of subjects with MDD, BD, and psychically healthy controls and show herein that (1) significantly reduced densities of GS-immunoreactive glial cells occur in cortical areas of MDD, but not BD subjects and (2) these changes are restricted to GS expressing ACs, whereas GS expressing OLs are normal in mood disorders.

## Materials and Methods

### Subjects

All brains were obtained from the New Magdeburg brain collection. Case recruitment, acquisition of personal data, performance of autopsy, and handling of autoptic material were conducted in strict accordance with the Declaration of Helsinki, and have been approved by the responsible Ethical Committee of Magdeburg. Written consent was obtained from the next-of-kin. Information for clinical diagnoses was obtained from clinical records and/or structural interviews of physicians involved in treatment or relatives (Bielau et al., 2005).

Brains of 29 human subjects with mood disorders according to DSM-IV were studied. Of these individuals 14 (8 female, 6 male; mean age: 46.9 ± 11.4 years) had suffered from a MDD and 15 (5 female, 10 male; mean age: 53.5 ± 10.4 years) had a BD. Sixteen control individuals (9 female, 7 male; mean age: 50.4 ± 11.0 years) without a history of neuropsychiatric disorder were also investigated. None of the patients or controls had a history of substance abuse or alcoholism. Neuropathological changes due to neurodegenerative or traumatic processes were ruled out by an experienced neuropathologist as previously described (Bielau et al., 2005). These cases were matched with respect to age, gender, and autolysis time. The matching processes were done prior to all analyses. For demographical, clinical, and psychopharmacological details see **Tables 1** and **2**. The mean daily doses of psychotropic medication taken by patients during the last 90 lifetime days were established according to the clinical files. Since our brain bank has been established about 25 years ago, patients received tricyclic antidepressants instead of selective serotonin or noradrenalin reuptake inhibitors (for detailed considerations, see Mosebach et al., 2013).

**Table 1:** Demographical data of patients and controls.

Diagnosis case	Gender	Age (years)	Postmortem autolysis time (h)	Duration of illness/years	Cause of death
**MDD (*n* = 14)**			
1	Male	42	5	n.a.	Suicide (hanging)
2	Female	39	48	7	Suicide (benzodiazepines overdose)
3	Female	46	48	11	Suicide (hanging)
4	Female	53	48	n.a.	Suicide (hanging)
5	Female	63	17	2	Pulmonary embolism
6	Female	61	70	11	Heart failure
8	Male	35	24	2	Suicide (slitting of the wrists)
9	Male	36	48	1	Suicide (hanging)
10	Male	42	24	n.a.	Acute pancreatitis
11	Male	30	24	n.a.	Suicide (hanging)
12	Female	60	24	1	Suicide (hanging)
13	Female	59	48	4	Suicide (hanging)
14	Female	35	24	n.a.	Suicide (strangulation)
15	Male	55	24	n.a.	Suicide (strangulation)
	**8 Female/6 Male**	**46.9 ± 11.4**	**34.0 ± 17.6**	**4.9 ± 4.3**	
**BD (*n* = 15)**			
16	Male	47	24	9	Suicide (stabbing)
17	Female	46	4	13	Suicide (tablet intoxication)
18	Male	42	12	16	Suicide (hanging)
19	Female	62	72	11	Pulmonary embolism
21	Male	39	24	2	Pulmonary embolism
22	Female	59	72	24	Suicide (tablet intoxication)
23	Male	39	56	14	Myocardial infarction
24	Male	69	48	26	Pulmonary embolism
25	Male	69	24	18	Heart failure, pulmonary embolism
26	Female	52	24	16	Heart failure, pulmonary embolism
27	Female	65	52	25	Heart failure
28	Male	44	96	6	Trombosis after myocardial infarction
29	Male	57	48	n.a.	Suicide (strangulation)
30	Male	60	24	5	Suicide (strangulation)
31	Male	53	24	1	Suicide (strangulation)
	**5 female/10 Male**	**53.5 ± 10.4**	**40.3 ± 25.8**	**13.3 ± 8.2**	
**Depression**	**13 female/16 Male**	**50.3 ± 11.2**	**37.2 ± 22.1**	**10.2 ± 8.1**	
**Controls (*n* = 16)**			
31	Male	56	48	0	Retroperitoneal hemorrhage
34	Female	52	24	0	Heart failure, renal insufficiency
35	Female	48	48	0	Status asthmaticus
38	Female	33	72	0	Aortic embolism
39	Female	50	72	0	Ruptured aortic aneurysm
40	Male	40	96	0	Myocardial infarction
41	Male	64	36	0	Ruptured aortic aneurysm
42	Female	48	26	0	Pulmonary embolism
43	Male	56	24	0	Myocardial infarction
44	Female	65	24	0	Heart failure
45	Female	30	48	0	Pulmonary embolism
46	Male	63	48	0	Heart failure (after heart surgery)
47	Female	38	24	0	Heart failure
48	Male	54	24	0	Pulmonary embolism
49	Male	46	24	0	Heart failure, cancer
50	Female	63	24	0	Myocardial infarction
	**9 Female/7 Male**	**50.4 ± 11.0**	**41.4 ± 22.2**	**-**	

**Table 2:** Psychopharmacological treatment.

Case	Mean antidepressiva dose of the last days [mg]	Mean neuroleptics dose of the last days [mg]	Mean benzodiazepine dose of the last days [mg]	Mean carbamazepine dose of the last days [mg]	Mean lithium dose of the last days [mg]
**MDD (*n* = 14)**			
1	n.a.	n.a.	n.a.	n.a.	n.a.
2	93	0	3	0	560
3	124	109	0	0	0
4	0	0	0	0	0
5	50	0	0	0	0
6	30	111	16	0	0
8	0	0	0	0	0
9	0	0	0	0	0
10	200	200	n.a.	n.a.	n.a.
11	100	100	n.a.	n.a.	n.a.
12	100	440	0	0	0
13	n.a.	n.a.	n.a.	n.a.	n.a.
14	n.a.	n.a.	n.a.	n.a.	n.a.
**BD (*n* = 15)**			
16	20	0	0	0	0
17	133	327	3	0	558
18	95	47	18	0	565
19	0	110	18	0	0
21	0	280	0	0	0
22	112	0	10	600	0
23	0	221	1	0	740
24	0	0	7	0	0
25	0	0	2	0	280
26	0	n.a.	n.a.	n.a.	n.a.
27	93	117	4	0	0
28	n.a.	n.a.	n.a.	n.a.	n.a.
29	n.a.	n.a.	n.a.	n.a.	n.a.
30	n.a.	n.a.	n.a.	n.a.	n.a.
31	150	200	0	200	0

### Tissue Processing

Brains were removed within 4–96 h after death and fixed in toto in 8% phosphate-buffered formaldehyde for at least 2 months (pH = 7.0, *T* = 15–20°C).

Frontal and occipital poles were separated by coronal cuts 0.9 cm anterior to the genu and posterior to the splenium of the corpus callosum. After embedding of all parts of the brains in paraffin, serial coronal sections of the prefrontal and the middle blocks were cut (20 μm) and mounted. The shrinkage factor caused by fixation and embedding of the brains was calculated by a method described previously (Bernstein et al., 1998a). The mean volume shrinkage factor for patients with affective disorders and controls was 2.21. No significant differences in the shrinkage factors among the three groups MDD, BP, and controls were found. Every 50th section was Nissl and myelin stained as described previously (Bernstein et al., 1998b).

### Glutamine Synthetase Immunohistochemistry

For immunohistochemical stainings, whole brain sections were collected at intervals of about 0.2 cm between 1.8 and 1 cm rostral to the genu of the corpus callosum. The pACC, (Brodmann Area 32) and dorsolateral prefrontal (DLPFC, Brodmann Area 9) cortices were easily identifiable using the “Atlas of the Human Brain” by Mai et al. (2003). Sections containing the left and right sACC, Aic, (Brodmann area 14), and the NAc were selected at intervals of 0.2 cm. To immunolocalize GS, we employed a well-characterized, monospecific polyclonal antiserum generated in rabbits against human GS (Prestige Antibody HPA 007316; Lot C 81287; from Sigma–Aldrich, Munich, Germany). Since different lots of the same antibody may considerably differ with regard to their staining properties (Couchman, 2009), we tested three different lots of the GS antiserum HPA 007316 (namely A42599, C81287, R04375). In our hands all three lots were of the same superior quality, and we decided to continue working with Lot C 81287. After dewaxing antigen demasking was carried out by boiling the sections for 4 min in 10 mM citrate buffer (pH 6.0). Thereafter, the sections were pre-incubated with methanol/H_2_O_2_ to suppress endogenous peroxidases and repeatedly washed with PBS. Subsequently, the primary GS antibody was applied at a dilution of 1:500 for 72 h at 4°C. Sections were then incubated with a biotinylated anti rabbit IgG (Amersham Bioscience, Buckinghamshire, GB), followed by the streptavidin horse radish complex for the application of the streptavidin–biotin technique (Amersham). The chromogen 3,3′-diaminobenzidine was used to visualize the reaction product. Subsequently, ammonium nickel sulfate hexahydrate was added to enhance the immunoreaction (Bernstein et al., 2013). For control purposes, the primary antiserum was replaced by either buffer or normal serum. Further control experiments involved the application of the GS antiserum after preabsorption with GS protein (recombinant human GS, charge number CE02; from Novoprotein, Shanghai, China) as described earlier in detail (Bernstein et al., 2014). When these controls were done the investigated regions did not show any specific immunostaining.

### Glial Fibrillary Acidic Protein (GFAP) Immunohistochemistry

For reasons of comparison and better delineation of cortical gray matter areas sections adjacent to GS immunostained ones were immunolabeled for GFAP. A monoclonal antibody (diluted 1:100 in PBS, from DAKO) was used. The secondary antibody was an anti-mouse peroxidase (from Biozol, Eching, Germany; dilution 1:50). The working dilution was 1:2000. Visualization was as described for GS. Controls involved replacement of the primary antiserum by either buffer or normal serum.

### Cell Countings

The actual section thickness after the histological procedures was 18.9 ± 1.0 μm (mean ± SD). The optical disector cell-counting method was employed. Cell countings were performed in two coronal sections per brain area under blind conditions. A counting grid was used to define a three-dimensional box within the thickness of the section as described previously (Bernstein et al., 1998b) allowing at least 4 μM guard zones at the top and bottom of the section, and to apply a direct, three-dimensional counting method. Fifteen consecutive boxes per left and right cortical area were positioned, spanning the layers I and II, layer III, and another 15 spanning the layers IV, V, and VI, thus subdividing cortical gray matter regions into superficial and deep layers as proposed by Katsel et al. (2011). To count immunostained cells in the NAc we used 15 boxes per section and hemisphere. The packing densities of glial cells are noted as the number of cells × 10^3^/mm^3^. The product of the volume and glial cell density provided the total cell number estimates. The inter-rater reliability was about 0.87. In a few cases we observed some local tissue damage in the brain region of interest. Cell counts of these particular regions were rigorously excluded from further calculations, which resulted in smaller cohorts than nominally indicated (for example, number of right NAc in MDD subjects: 12 instead of 14 cases).

### Statistical Analysis

A single-factor analysis of variance was performed using diagnostic groups as a three-level independent variable (MDD patients versus BD versus non-psychiatric controls) and measured and calculated parameters were treated as dependent variables. MANOVA was performed with diagnosis and side, ie., left and right hemisphere, as independent variables (repeated measures). Effect sizes were determined for 3-group comparisons (major depressive disorder, BD, controls). Confounding variables including whole brain volume were primarily tested on normality by use of the Kolmogorov–Smirnov test. A *post hoc* Tukey HSD test was conducted to determine which variables were significantly different from each other. Pearson correlation tests were carried out to investigate effects of postmortem delay, time of fixation, illness duration, number of illness episodes, and psychotropic medication (i.e., antidepressants, neuroleptics, benzodiazepines, and lithium) on data. In addition, emphasis was given to suicide as possible confounding factor. *P*-values less than 0.05 were deemed to be statistically significant.

## Results

### Qualitative Observations

#### Glutamine Synthetase Immunostaining

##### Astrocytes

Glutamine synthetase -immunoreactive ACs were abundantly present in the cerebral cortex and the NAc. Their morphology was remarkably consistent. Astrocytic somata and processes were prominently stained for GS. In addition, an intense immunostaining was observed in the neuropil. Numerous blood vessels were surrounded by GS-immunoreactive AC endfeet.

##### Oligodendrocytes

**FIGURE 1:**
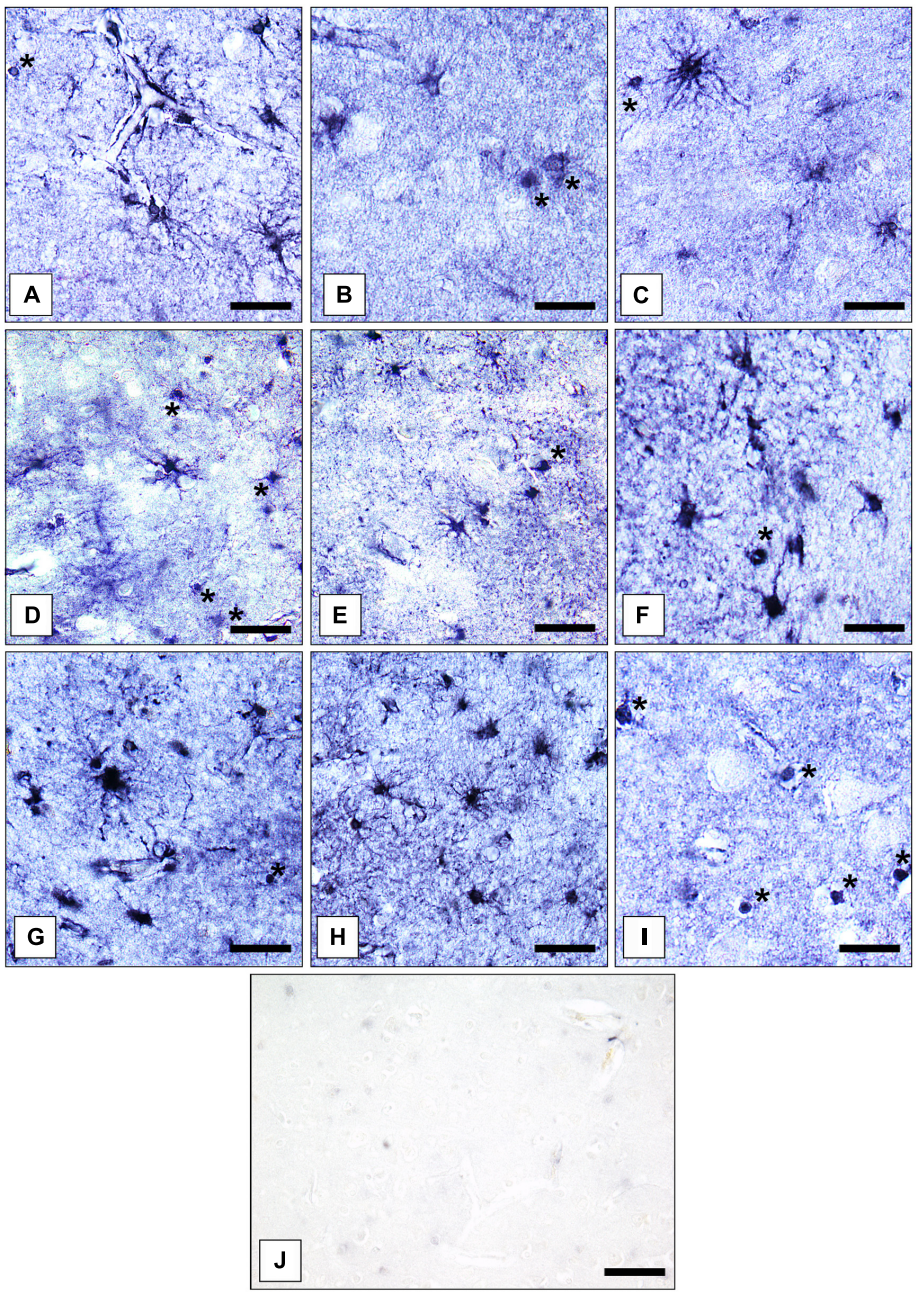
**Immunolocalization of GS in cortical and subcortical human brain glial cells in MDD, BD, and controls. (A)** GS-immunoreactive ACs and OLs (asterisk) in the pACC (control case). Bar = 20 μm. **(B)** GS-immunoreactive ACs and OLs (asterisks) in the pACC (MDD subject). Bar = 20 μm. **(C)** GS-immunoreactive ACs and OLs (asterisk) in the pACC (BD subject). Bar = 20 μm. **(D)** GS-immunoreactive ACs and OLs (asterisks) in the sACC (control case). Bar = 20 μm.**(E)** GS-expressing ACs and OLs (asterisk) in the sACC (MDD subject). Bar = 20 μm.**(F)** GS-expressing ACs and OLs (asterisk) in the DLPFC (BD subject). Bar = 20 μm. **(G)** GS-immunopositive ACs in the AiC (control case). Bar = 20 μm. **(H)** GS-expressing ACs in the NAc (control case). Bar = 24 μm. **(I)** GS-immunoreactive OLs (asterisks) in the pACC (control case). Bar = 20 μm. **(J)** Specificity control reaction. After preabsorption of the primary antiserum with recombinant GS protein no specific immunostaining is visible. Bar = 30 μm.

Gray matter GS-immunoreactive OLs were easily identifiable based on their typical morphology. The immunoreaction was confined to the cell somata. Short processes were only infrequently immunolabeled. Immunopositive OLs were fairly uniformly distributed throughout prefrontal cerebral cortex. However, in the AiC and the NAc GS-expressing OLs were relatively rarely found. Therefore, we did not count them separately in the latter two brain regions. Examples for the immunolocalization of GS in ACs and OLs are given in **Figures 1A–I**. **Figure 1J** shows a control reaction.

#### GFAP Immunostaining

In all cortical areas GFAP protein was expressed in a majority of ACs. Their distribution showed a laminar pattern. The highest package density of GFAP was found in layers I (where ACs abut at the pial surface of the brain, Miguel-Hidalgo et al., 2010) and II. In the NAc an even distribution of GFAP immunolabeled cell elements was observed. OLs did not express GFAP.

### Quantitative Estimates

We could replicate our own findings (Bernstein et al., 2014) as well as results previously published by others (Toro et al., 2006) that there is a higher density of GS-expressing cells in superficial cortical layers I–III than in deeper cortical layers IV–VI. It is therefore justified to count superficial and deeper layers separately.

**FIGURE 2:**
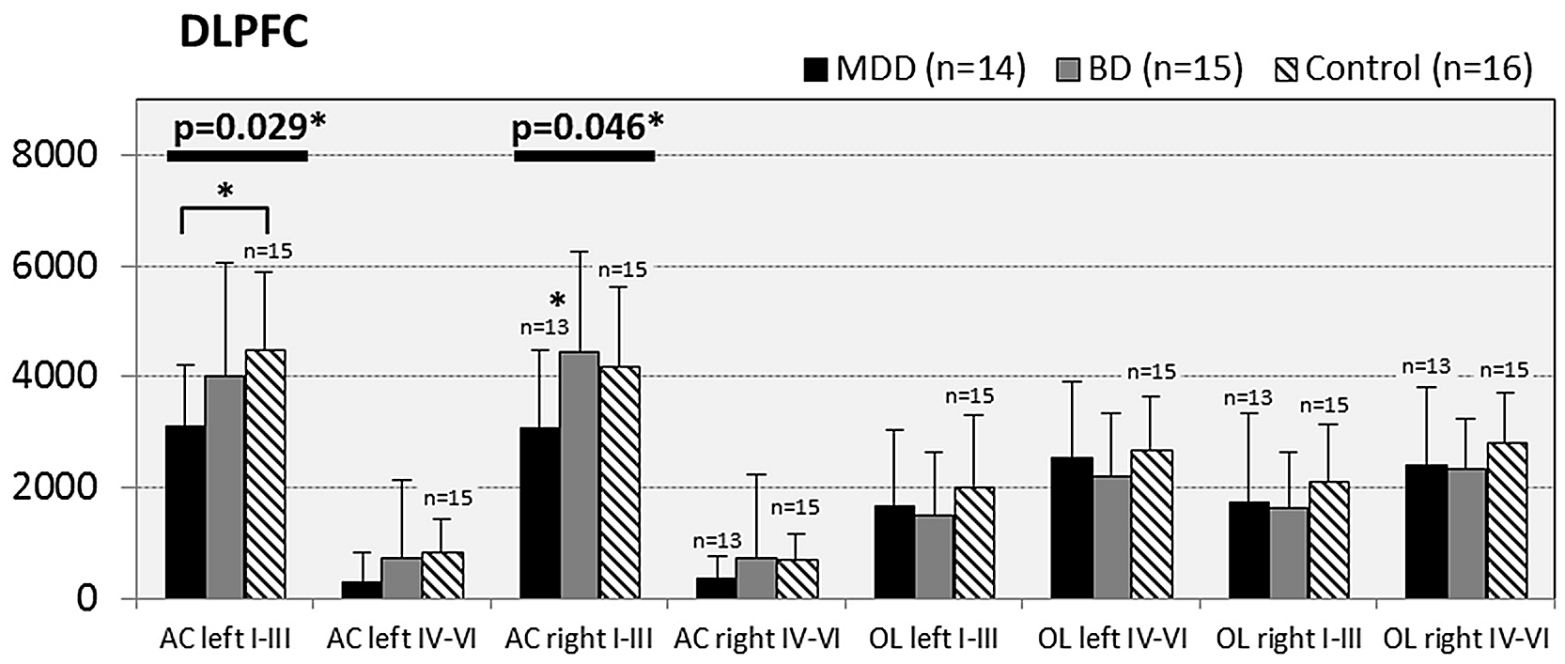
**Numerical densities of GS-expressing glial cells (ACs and OLs) in the DLPFC of subjects with MDD, BD, and controls.** Compared with controls the density of GS-expressing ACs is significantly reduced in MDD cases (AC left I–III, *p* = 0.029; AC right I–III, *p* = 0.046).

**FIGURE 3:**
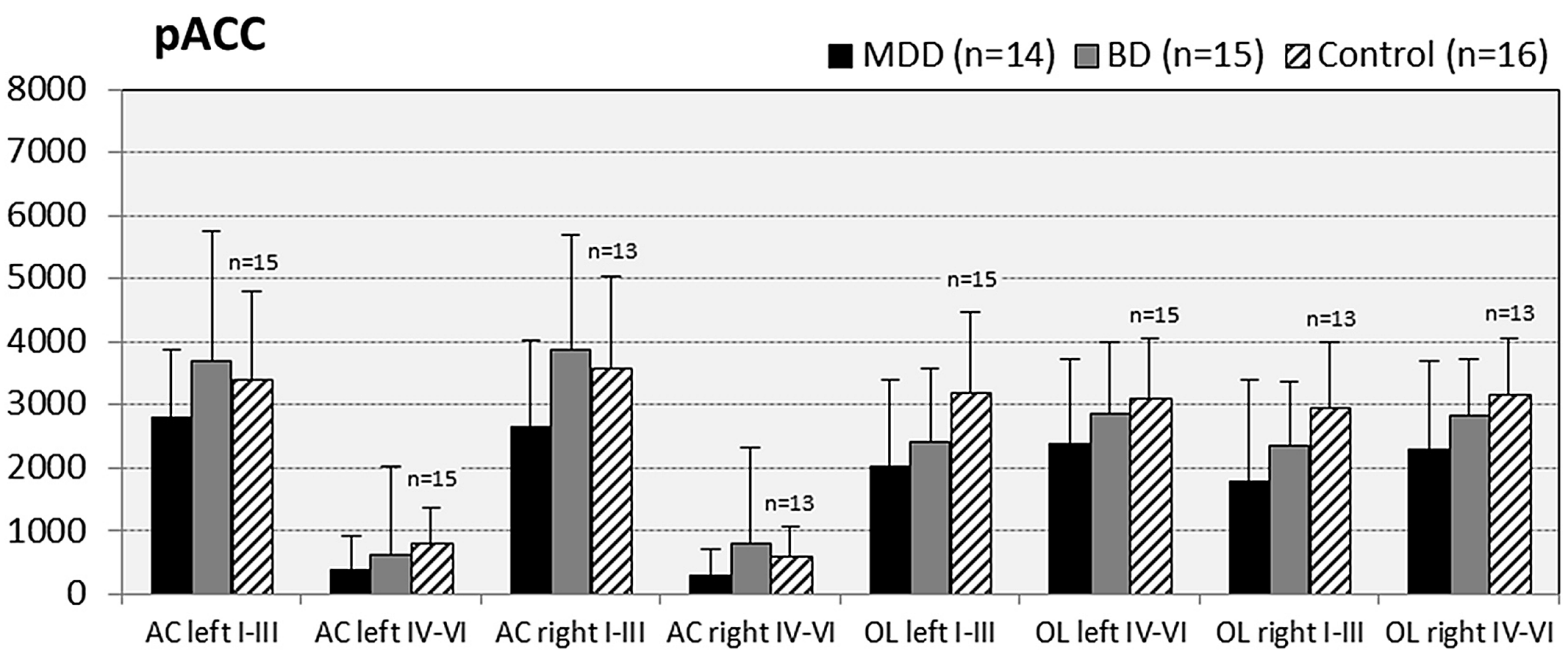
**Numerical densities of GS-expressing glial cells (ACs and OLs) in the pACC of subjects with MDD, BD, and controls.** No significant alterations were found in this brain region.

**FIGURE 4:**
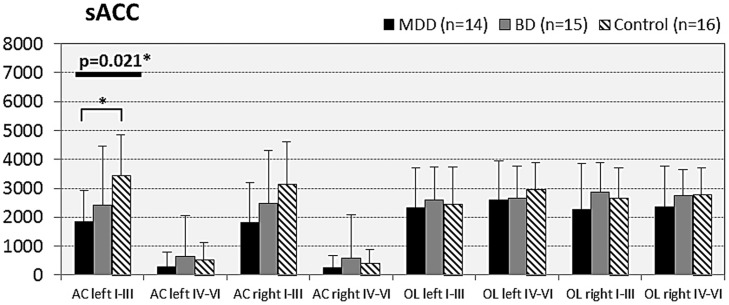
**Numerical densities of GS-expressing glial cells (ACs and OLs) in the sACC of subjects with MDD, BD, and controls.** Compared with controls the density of GS-expressing ACs is significantly reduced in MDD cases (AC left I–III, *p* = 0.021).

**FIGURE 5:**
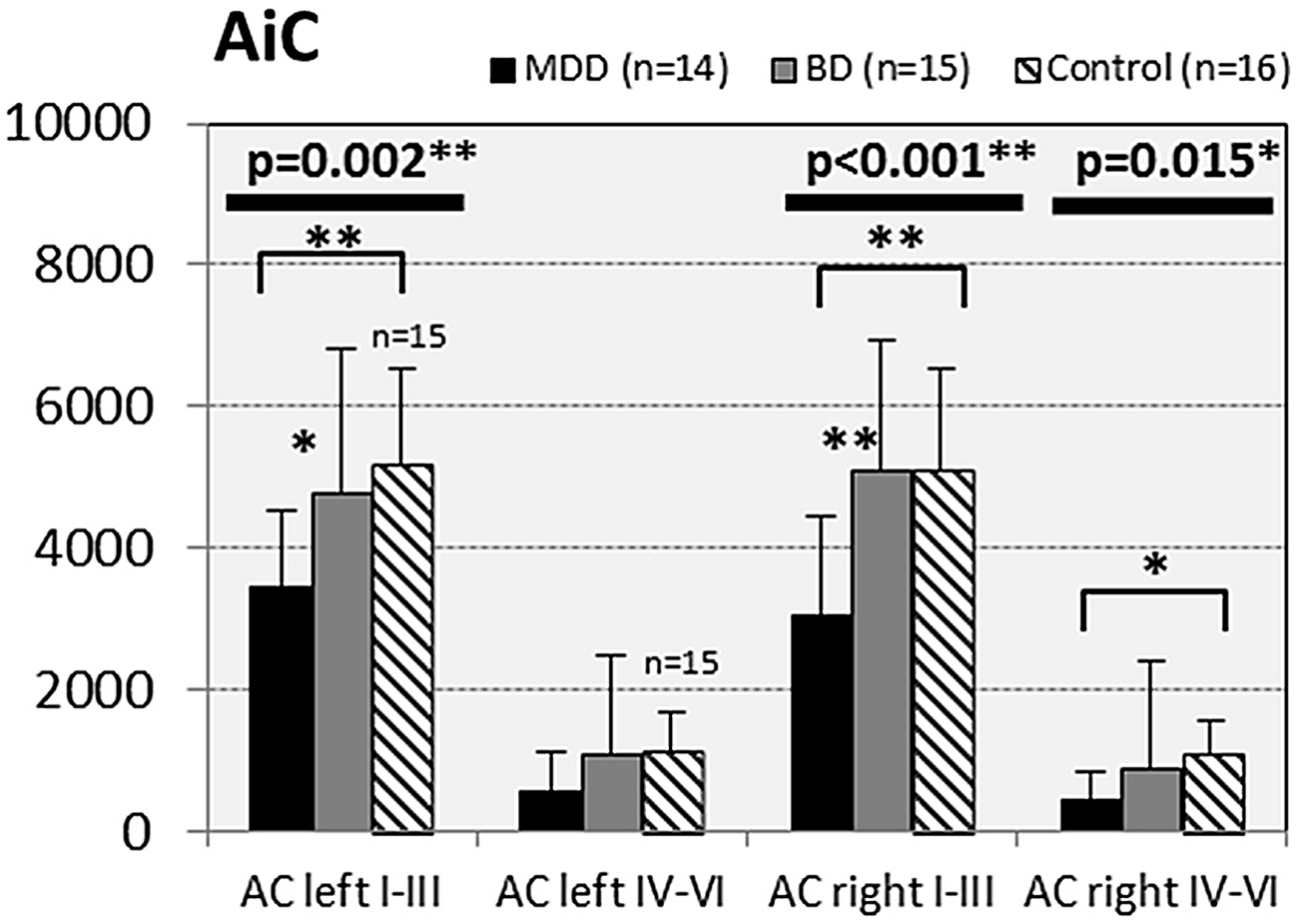
**Numerical densities of GS-expressing glial cells (ACs and OLs) in the AiC of subjects with MDD, BD, and controls.** Compared with controls the density of GS-expressing ACs is significantly reduced in MDD cases (AiC left I–III, *p* = 0.002; AiC right I–III, *p* = 0.001; AiC right IV–VI, *p* = 0.015).

**FIGURE 6:**
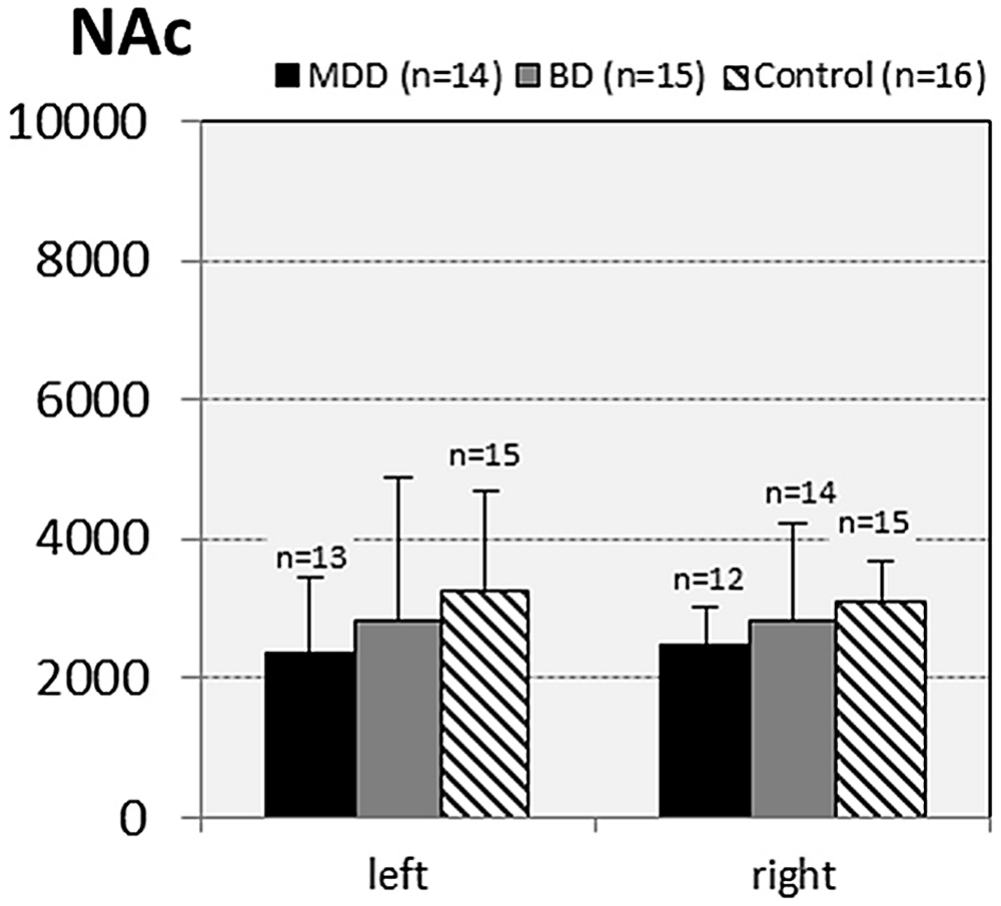
**Numerical densities of GS-expressing glial cells (ACs and OLs) in the NAc of subjects with MDD, BD, and controls.** No significant alterations were found in this brain region.

In subjects with mood disorder significantly reduced numerical densities of GS immunoreactive ACs were found the DLPFC (left side, layers I–III, *p* = 0.029; *F* = 4.487 and right side, layers I–III; *p* = 0.046; *F* = 3.647), sACC (left side, layers I–III, *p* = 0.021; *F* = 4.304), AiC (left side, layers I–III, *p* = 0.002; *F* = 7.893; right side, layers I–III, *p* = 0.001; *F* = 9.789; ride side, layers IV–VI, *p* = 0.015; *F* = 5.136). No significant changes were detectable in the pACC and the NAc. All but one (DLPCC right side, layers I–III) significant changes survived Tukey’s HSD *post hoc* tests. Notably, no significant alterations in the numerical densities of OLs were found. Results of the morphometric analyses are shown in **Figures 2**–**6**.

#### MDD vs. controls

Compared with controls a significant reduction in the numerical densities of GS-immunopositive ACs was found in MDD in five of the 10 cortical and subcortical brain regions studied: DLPFC (left side, layers I–III, *p* = 0.024), sACC (left side, layers I–III, *p* = 0.018); AiC (left side, layers I–III; *p* = 0.002; right side, layers I–III; *p* = 0.001; *F* = 9.962; right side, layers IV–VI, *p* = 0.012).

#### BD vs. controls

In BD cases the numerical densities of GS-expressing ACs and OLs did not significantly differ from those of controls.

#### MDD vs. BD

Compared with BD cases subjects with MDD showed significantly decreased densities of GS-immunopositive ACs in the DLPFC (right side, layers I–III, *P* = 0.047), in the AiC (left side, layers I–III, *p* = 0.024; right side, layers IV.IV; *p* = 0.001).

#### Impact of Completed Suicide on Cell Densities

**FIGURE 7:**
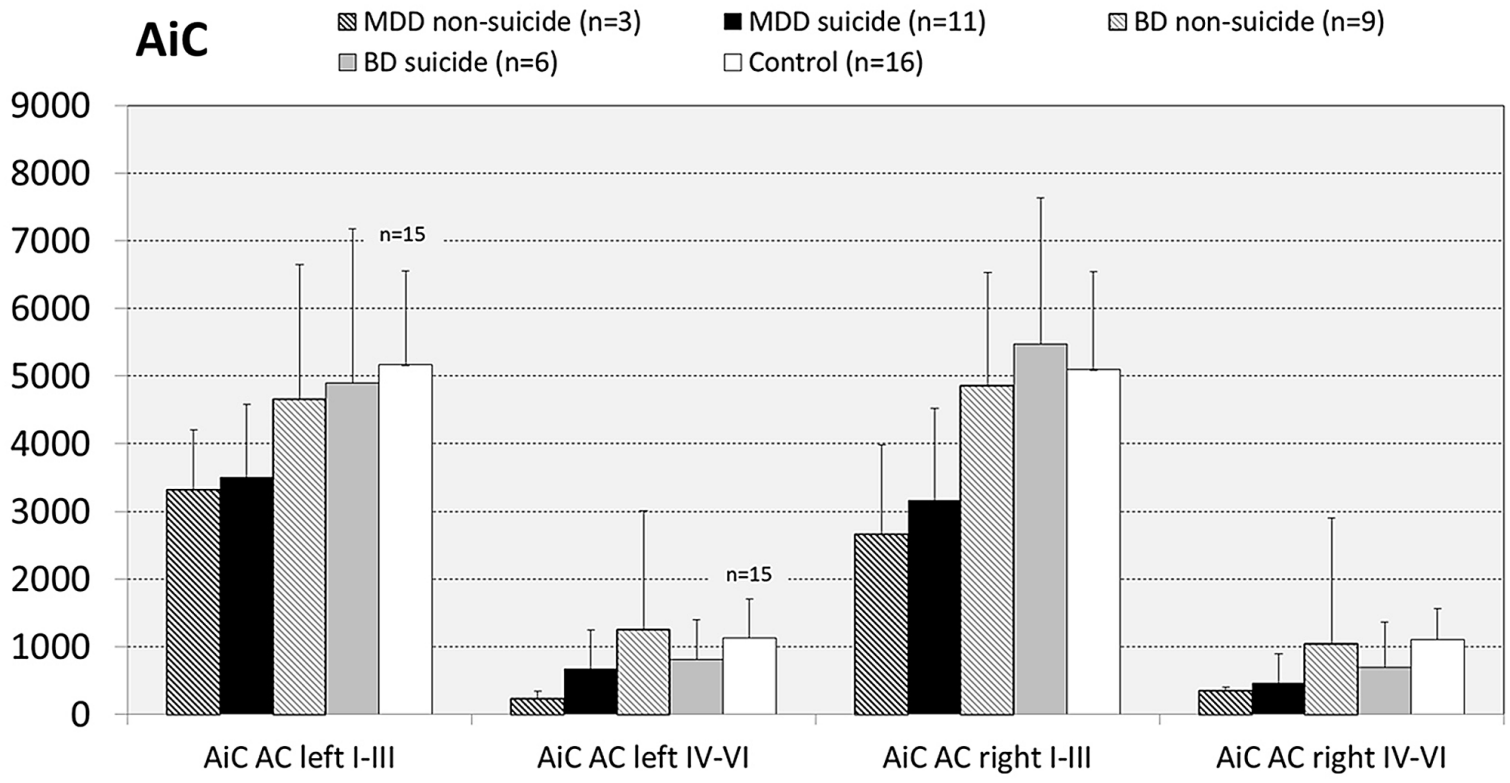
**Numerical densities of GS-expressing glial cells in the AiC of suicide completers with MDD, non-suicide completers with MDD, suicide completers with BD, non-suicide completers with BD, and controls.** No significant alterations were found.

Since there are reports showing that GS expression is altered in brains of suicide victims with and without mood disorder (Kim et al., 2007; Sequeira et al., 2009; Bernstein et al., 2013) we next analyzed the influence of the confounding factor “death by suicide” on the densities of GS immunoreactive ACs in the brain regions under study. For this purpose we divided MDD and BD cases into subgroups of suicide victims (MDD: *N* = 11; BD: *N* = 7) and depressed subjects who died of natural causes (MDD: *N* = 3; BD: *N* = 8). With regard to MDD cases in none of the brain regions there appeared significant differences in the densities of GS-expressing cells between depressed subjects dying by suicide and non-suicidal individuals with MDD. The same holds true for subjects with BD: Suicidal and non-suicidal BD cases did not significantly differ with regard to glial cell densities in any of the regions studied, as exemplified for the AiC in **Figure 7**. It should be emphasized, however, that the subgroup of non-suicidal subjects with MDD is too small (*N* = 3) to come to far-reaching conclusions from these data.

#### The Influence of Other Confounding Factors

Analysis of the potential confounding factors on the test results revealed no significant influence of age, gender, duration of disease, or psychotropic medication. Especially the lack of correlation between the age and the density of GS-expressing ACs is interesting, because Rajkowska and Stockmeier (2013) have hypothesized the glial pathology appears to apply mostly to younger and middle age subjects with MDD (<60 years of age).

#### Possible Influence of Hemispheric Asymmetry on GS Cell Distribution in Human Brain

There is evidence in the for structural, functional, and physiological asymmetries in the two hemispheres of human brain (comprehensively reviewed in Jayasundar and Raghunathan, 1997), which involve aspects of glutamate and GABA metabolism in health and mood disorder (MDD: Pfleiderer et al., 2003; Bajbouj et al., 2006; Gos et al., 2012; BD: Gos et al., 2012; Xu et al., 2013). The latter might in part result from left–right hemispheric differences in the distribution of GS immunoreactivity (Bernstein et al., 2013), Unfortunately, knowledge about the catalytic activity and distribution of human brain GS comes mainly from studies of only one (namely the left) brain hemisphere (Burbaeva et al., 2003; Rajkowska and Miguel-Hidalgo, 2007; Miguel-Hidalgo et al., 2010). To exclude a possible left–right asymmetry of GS, we counted left and right hemispheres separately. No evidence was found for a significant left–right asymmetry, neither in controls, nor in subjects with affective disorder.

## Discussion

Major depression disorder and BD are serious mental illnesses with multifactorial pathophysiologic characteristics. The past years have witnessed a remarkable extension of our understanding of the neurobiology of affective disorders, adding the glutamatergic and the GABAergic hypotheses to “classical” monoaminergic theories of mood dysregulation (Delgado, 2000; Skolnick et al., 2009; Catena-Dell’Osso et al., 2013; Dou et al., 2013; Hertz et al., 2014; Pehrson and Sanchez, 2015; Schitine et al., 2015). Besides other indicators of compromised amino acidergic neurotransmission in depression, two large meta-analyses of neuroimaging findings have demonstrated decreased levels of the Glx in adult MDD patients, whereas in the brains of BD patients increased, decreased, and unchanged glutamate and reduced glutamine levels have been measured (Yüksel and Öngür, 2010; Gigante et al., 2012; Luykx et al., 2012). Moreover, the NMDA receptor antagonist ketamine has been shown in clinical trials to act as a rapid antidepressant in MDD (for recent considerations, see DeWilde et al., 2015). The acceptance of a crucial role of glutamate in the pathology of mental disorders entails the obligatory implication of glial cells (Arnone et al., 2015). Being part of the glutamatergic tripartite synapse, intact ACs are central to a proper functioning of glutamatergic neurotransmission, and imbalances in the neuron-AC communication at the synaptic level might significantly contribute to affective disorders, such as mania and depression (Mitterauer, 2015; Thompson et al., 2015). The enzyme GS, which is highly expressed in, but in its expression not restricted to, ACs, is required to synthesize the non-toxic glutamine from the re-uptake of either glutamate or GABA. Besides, in human brain an immunologically and enzymatically very closely related “sister” enzyme of (GS-like protein) of unknown cellular localization was found (Boksha et al., 2000), which shows altered expressions in schizophrenia and Alzheimer’s disease (Burbaeva et al., 2003; Burbaeva et al., 2014). The possible implication of GS-like enzyme for mood disorders is unexplored, however. Keeping this in mind when designing this study, we morphometrically analyzed GS-immunoreactive glial cells located in various cortical and subcortical gray matter areas of subjects with MDD and BD, thereby counting, wherever possible, ACs and OLs separately. Significantly reduced densities of GS immunopositive ACs were found in the prefrontal areas DLPFC and sACC as well as in the AiC of subjects with MDD. Remarkably, the densities of GS-immunopositive OLs were normal in all regions studied, although a significant loss of perineuronal OLs was found in prefrontal cortex sublayers IIIa, b, and c in mood disorder by others (Vostrikov et al., 2007). When carefully analyzing cortical GS-expressing OLs in mice, Takasaki et al. (2010) found that the enzyme protein is mainly expressed in perineuronal OLs, with roughly half of them being GS-immunopositive. However, these GS-immunoreactive OLs lack plasmalemmal glutamate transporters GLAST and GLT-1, thus apparently being unable to participate in the glutamate-glutamine cycle. The reason why these cells even so express GS remains enigmatic, but is seemingly not directly related to glutamatergic synaptic neurotransmission (possibly playing a role in cellular ammonia clearance and/or certain metabolic support to the associating cortical neurons, Takasaki et al., 2010; Bernstein et al., 2014). Thus, our data clearly show a cell-type specific reduction in cortical GS expression in individuals with MDD, which cannot be revealed when analyzing total GS protein in brain samples with biochemical methods. Interestingly, we found no indication for altered densities of GS-expressing ACs in the NAc, although this brain region plays a central role in MDD (Thompson et al., 2015) and has therefore been chosen as a target structure for deep brain stimulation in cases of therapy-resistant MMD (Bewernick et al., 2012). Although ACs play a critical role in controlling the excitability of NAc neurons via activation of glutamatergic receptors (Fellin et al., 2007; Thompson et al., 2015), their possible implication for “human” depression” yet is poorly explored. However, the number of ACs in the NAc was found to be unchanged in a developmental “toxic stress” model of depression (Shende et al., 2015).

Unlike in MDD, no alterations in the density of GS-expressing glial cells were found in BD cases. This is in agreement with earlier findings by Toro et al. (2006), but in contrast to reduced GS expression in prefrontal area of BD subjects as reported by Choudary et al. (2005). Two possible conclusions may be drawn from our data: either (1) glia associated-GS is normal, because GS does not play a prominent role in the pathophysiology of BD, and/or (2) GS expression was “normalized” (most probably up-regulated) by long-term treatment of the disease. Given the latter possibility, two putative factors might have increased GS expression in brains of BD subjects: administration of lithium (Kalkman, 2011) and treatment with antidepressants (Hashioka et al., 2013; Liu et al., 2015). Our calculations, however, did not reveal significant positive correlations of numerical densities of GS expressing glial cells and either of the aforementioned two factors. Hence we tend to believe that GS is not a major contributing factor to BD neuropathology. It should be stated, however, that disease-related alterations in the cerebral expression of GS do not always exert a direct influence on the glutamate–glutamine–GABA ratio. So, for example, a strong upregulation of GS protein was reported for the anterior cingulate cortex of female but not male individuals with schizophrenia (Martins-de-Souza et al., 2010), whereas no such gender-specific change in the Glx ratio was found in schizophrenia patients by MRS (Rowland et al., 2013). Thus, further studies are clearly needed to learn more about the impact of GS on glutamate metabolites in the brain.

### Limitations of the Study

Since a major limitation of post-mortem studies is underpowered sample size, we have tried to increase the three cohorts (controls, *N* = 16; BD, *N* = 15, MDD, *N* = 14). A consequence from doing so is to accept the lack of data on the cumulative antidepressant exposure of some patients with MDD and BD. A further limitation of the this study is that, as in all immunocytochemistry-based morphometric studies, it cannot be said with ultimate certainty, whether the observed disease-related changes are due to glial cell loss, or to reduced intracellular expression of the protein (below the detection threshold of the method) in still existing cells, or both. Lastly, a limitation may arise from the fact that the decreased expression of GS protein does not necessarily mean reduced activity of the enzyme. Unfortunately, it is impossible to reveal the catalytic activity of the AC-located GS enzyme because of the lack of a specific enzyme histochemical method for GS.

## Conclusion

In MDD but not in BD there is a glia cell-type specific (astroglial) reduction of cortical GS protein expression, which might constitute a cellular correlate of lower cortical Glx levels reported for subjects with MDD in MSR studies.

## Author Contributions

H-GB analyzed the data, researched, wrote, and edited the manuscript. GM-L analyzed the data. HD carried out statistical calculations and contributed to photography. JB analyzed the data. JS wrote, and edited the manuscript. MW wrote and edited the manuscript. BB wrote and edited the manuscript.

## Conflict of Interest Statement

The authors declare that the research was conducted in the absence of any commercial or financial relationships that could be construed as a potential conflict of interest.

## Abbreviations

AC(s): astrocyte(s)
Aic: anterior insular cortex
ANOVA: A single-factor analysis of variance
ATP: adenosine triphosphate
BD: bipolar disorder
CNS: Central Nervous System
DLPFC: dorso-lateral prefrontal cortex
GABA: γ-Aminobutyric acid
DSM-IV: Diagnostic and Statistical Manual of Mental Disorders IVth edition
GFAP: glial fibrillary acidic protein
Glx: glutamate-glutamine-GABA complex
GS: glutamine synthetase
MANOVA: Multivariate analysis of covariance
MDD: major depression disorder
MSR: Magnetic Resonance Spectroscopy
OL(s): oligodendrocyte(s)
n.a.: not available
NAc: Nuc. Accumbens
pACC: pregenual anterior cingulate
PBS: Phosphate buffered saline
sACC: subgenual anterior cingulate cortex

## References

[B1] Altshuler L. L. Abulseoud O. A. Foland-Ross L. Bartzokis G. Chang S. Mintz J. (2010). Amygdala astrocyte reduction in subjects with major depression but not bipolar disorder. *Bipolar Disord.* 1 451–549. 10.1111/j.1399-5618.2010.00838.x 20712756

[B2] Anlauf E. Derouiche A. (2013). Glutamine synthetase as an astrocytic marker: its cell type and vesicle localization. *Front. Endocrinol.* 4:144 10.3389/fendo.2013.00144 PMC3797418 24137157

[B3] Arnone D. Mumuni A. N. Jauhar S. Condon B. Cavanagh J. (2015). Indirect evidence of selective glial involvement in glutamate-based mechanisms of mood regulation in depression: meta-analysis of absolute prefrontal neuro-metabolic concentrations. *Eur. Neuropsychopharmacol.* 10.1016/j.euroneuro.2015.04.016 [Epub ahead of print]. 26028038

[B4] Bajbouj M. Lisanby S. H. Lang U. E. Danker-Hopfe H. Heuser I. Neu P. (2006). Evidence for impaired cortical inhibition in patients with unipolar major depression. *Biol. Psychiatry* 59 395–400. 10.1016/j.biopsych.2005.07.036 16197927

[B5] Barley K. Dracheva S. Byne W. (2009). Subcortical oligodendrocyte-and astrocyte-associated gene expression in subjects with schizophrenia, major depression and bipolar disorder. *Schizophr. Res.* 112 54–64. 10.1016/j.schres.2009.04.01 19447584

[B6] Beasley C. L. Pennington K. Behan A. Wait R. Dunn M. J. Cotter D. (2006). Proteomic analysis of the anterior cingulate cortex in the major psychiatric disorders: evidence for disease-associated changes. *Proteomics* 6 3414–3425. 10.1002/pmic.200500069 16637010

[B7] Bernstein H. G. Bannier J. Meyer-Lotz G. Steiner J. Keilhoff G. Dobrowolny H. (2014). Distribution of immunoreactive glutamine synthetase in the adult human and mouse brain. Qualitative and quantitative observations with special emphasis on extra-astroglial protein localization. *J. Chem. Neuroanat.* 61–62, 33–50. 10.1016/j.jchemneu.2014.07.003 25058171

[B8] Bernstein H. G. Krell D. Baumann B. Danos P. Falkai P. Diekmann S. (1998a). Morphometric studies of the entorhinal cortex in neuropsychiatric patients and controls: clusters of heterotopically displaced lamina II neurons are not indicative of schizophrenia. *Schizophr. Res.* 33 125–132. 10.1016/S0920-9964(98)00071-1 9789904

[B9] Bernstein H. G. Stanarius A. Baumann B. Henning H. Krell D. Danos P. (1998b). Nitric oxide synthase-containing neurons in the human hypothalamus: reduced number of immunoreactive cells in the paraventricular nucleus of depressive patients and schizophrenics. *Neuroscience* 83 867–875. 10.1016/S0306-4522(97)00461-2 9483570

[B10] Bernstein H. G. Steiner J. Guest P. C. Dobrowolny H. Bogerts B. (2015). Glial cells as key players in schizophrenia pathology: recent insights and concepts of therapy. *Schizophr. Res.* 161 4–18. 10.1016/j.schres.2014.03.035 24948484

[B11] Bernstein H. G. Tausch A. Wagner R. Steiner J. Seeleke P. Walter M. (2013). Disruption of glutamate-glutamine-GABA cycle significantly impacts on suicidal behaviour: survey of the literature and own findings on glutamine synthetase. *CNS Neurol. Disord. Drug Targets* 12 900–913. 10.2174/18715273113129990091 24040807

[B12] Bewernick B. H. Kayser S. Sturm V. Schlaepfer T. E. (2012). Long-term effects of nucleus accumbens deep brain stimulation in treatment-resistant depression: evidence for sustained efficacy. *Neuropsychopharmacology* 37 1975–1985. 10.1038/npp.2012.44 22473055 PMC3398749

[B13] Bielau H. Trübner K. Krell D. Agelink M. W. Bernstein H. G. Stauch R. (2005). Volume deficits of subcortical nuclei in mood disorders A postmortem study. *Eur. Arch. Psychiatry Clin. Neurosci.* 255 401–412. 10.1007/s00406-005-0581-y 16080015

[B14] Boksha I. S. Tereshkina E. B. Burbaeva G. S. (2000). Glutamine synthetase and glutamine synthetase-like protein from human brain: purification and comparative characterization. *J. Neurochem.* 75 2574–2582. 10.1046/j.1471-4159.2000.0752574.x 11080211

[B15] Brennan B. P. Hudson J. I. Jensen J. E. McCarthy J. Roberts J. L. Prescot A. P. (2010). Rapid enhancement of glutamatergic neurotransmission in bipolar depression following treatment with riluzole. *Neuropsychopharmacology* 35 834–846. 10.1038/npp.2009.191 19956089 PMC3055603

[B16] Burbaeva G. Sh. Boksha I. S. Tereshkina E. B. Savushkina O. K. Prokhorova T. A. Vorobyeva E. A. (2014). Glutamate and GABA-metabolizing enzymes in post-mortem cerebellum in Alzheimer’s disease: phosphate-activated glutaminase and glutamic acid decarboxylase. *Cerebellum* 13 607–615. 10.1007/s12311-014-0573-4 24950944

[B17] Burbaeva G. Sh. Boksha I. S. Turishcheva M. S. Vorobyeva E. A. Savushkina O. K. Tereshkina E. B. (2003). Glutamine synthetase and glutamate dehydrogenase in the prefrontal cortex of patients with schizophrenia. *Prog. Neuropsychopharmacol. Biol. Psychiatry.* 27 675–680. 10.1016/S0278-5846(03)00078-2 12787856

[B18] Catena-Dell’Osso M. Fagiolini A. Rotella F. Baroni S. Marazziti D. (2013). Glutamate system as target for development of novel antidepressants. *CNS Spectr.* 18 188–198. 10.1017/S1092852912000971 23369807

[B19] Choudary P. V. Molnar M. Evans S. J. Tomita H. Li J. Z. Vawter M. P. (2005). Altered cortical glutamatergic and GABAergic signal transmission with glial involvement in depression. *Proc. Natl. Acad. Sci. U.S.A.* 102 15653–15658. 10.1073/pnas.0507901102 16230605 PMC1257393

[B20] Cotter D. Mackay D. Chana G. Beasley C. Landau S. Everall I. P. (2002). Reduced neuronal size and glial cell density in area 9 of the dorsolateral prefrontal cortex in subjects with major depressive disorder. *Cereb. Cortex* 12 386–394. 10.1093/cercor/12.4.386 11884354

[B21] Cotter D. Mackay D. Landau S. Kerwin R. Everall I. (2001a). Reduced glial cell density and neuronal size in the anterior cingulate cortex in major depressive disorder. *Arch. Gen. Psychiatry* 58 545–553. 10.1001/archpsyc.58.6.545 11386983

[B22] Cotter D. R. Pariante C. M. Everall I. P. (2001b). Glial cell abnormalities in major psychiatric disorders: the evidence and implications. *Brain Res. Bull.* 55 585–595. 10.1016/S0361-9230(01)00527-5 11576755

[B23] Couchman J. R. (2009). Commercial antibodies: the good, bad, and really ugly. *J. Histochem. Cytochem.* 57 7–8. 10.1369/jhc.2008.952820 18854593 PMC2605718

[B24] Davis S. Thomas A. Perry R. Oakley A. Kalaria R. N. O’Brien J. T. (2002). Glial fibrillary acidic protein in late life major depressive disorder: an immunocytochemical study. *J. Neurol. Neurosurg. Psychiatry* 73 556–560. 10.1136/jnnp.73.5.556 12397151 PMC1738142

[B25] Delgado P. L. (2000). Depression: the case for a monoamine deficiency. *J. Clin. Psychiatry* 61(Suppl. 6), 7–11. 10775018

[B26] DeWilde K. E. Levitch C. F. Murrough J. W. Mathew S. J. Iosifescu D. V. (2015). The promise of ketamine for treatment-resistant depression: current evidence and future directions. *Ann. N. Y. Acad. Sci.* 1345 47–58. 10.1111/nyas.12646 25649308 PMC4447578

[B27] Dong X. H. Zhen X. C. (2015). Glial pathology in bipolar disorder: potential therapeutic implications. *CNS Neurosci. Ther.* 21 393–397. 10.1111/cns.12390 25753128 PMC6495495

[B28] Dou W. Palomero-Gallagher N. van Tol M. J. Kaufmann J. Zhong K. Bernstein H. G. (2013). Systematic regional variations of GABA, glutamine, and glutamate concentrations follow receptor fingerprints of human cingulate cortex. *J. Neurosci.* 33 12698–12704. 10.1523/JNEUROSCI.1758-13.20 23904606 PMC6618546

[B29] Duman R. S. (2014). Neurobiology of stress, depression, and rapid acting antidepressants: remodeling synaptic connections. *Depress. Anxiety* 31 291–296. 10.1002/da.22227 24616149 PMC4432471

[B30] Duncan L. E. Holmans P. A. Lee P. H. O’Dushlaine C. T. Kirby A. W. Smoller J. W. (2014). Pathway analyses implicate glial cells in schizophrenia. *PLoS ONE* 9:e89441 10.1371/journal.pone.0089441 PMC3933626 24586781

[B31] Etiévant A. Lambás-Señas L. Scarna H. Lucas G. Haddjeri N. (2013). Astrocytes and gliotransmitters: new players in the treatment of major depression? *Curr. Drug Targets* 14 1295–1307. 10.2174/13894501113149990197 24010966

[B32] Fellin T. D’Ascenzo M. Haydon P. G. (2007). Astrocytes control neuronal excitability in the nucleus accumbens. *ScientificWorldJournal* 7 89–97. 10.1100/tsw.2007.195 17982581 PMC5901197

[B33] Gigante A. D. Bond D. J. Lafer B. Lam R. W. Young L. T. Yatham L. N. (2012). Brain glutamate levels measured by magnetic resonance spectroscopy in patients with bipolar disorder: a meta-analysis. *Bipolar Disord.* 14 478–487. 10.1111/j.1399-5618.2012.01033.x 22834460

[B34] Gos T. Schroeter M. L. Lessel W. Bernstein H. G. Dobrowolny H. Schiltz K. (2013). S100B-immunopositive astrocytes and oligodendrocytes in the hippocampus are differentially aﬄicted in unipolar and bipolar depression: a postmortem study. *J. Psychiatr. Res.* 47 1694–1699. 10.1016/j.jpsychires.2013.07.005 23896207

[B35] Gos T. Steiner J. Bielau H. Dobrowoln H. Günther K. Mawrin C. (2012). Differences between unipolar and bipolar I depression in the quantitative analysis of glutamic acid decarboxylase-immunoreactive neuropil. *Eur. Arch. Psychiatry Clin. Neurosci.* 262 647–655. 10.1007/s00406-012-0315-x 22526728 PMC3491185

[B36] Hamidi M. Drevets W. C. Price J. L. (2004). Glial reduction in amygdala in major depressive disorder is due to oligodendrocytes. *Biol. Psychiatry* 55 563–569. 10.1016/j.biopsych.2003.11.006 15013824

[B37] Harrison P. J. (2002). The neuropathology of primary mood disorder. *Brain* 125 1428–1449. 10.1093/brain/awf149 12076995

[B38] Hashioka S. Miyaoka T. Wake R. Furuya M. Horiguchi J. (2013). Glia: an important target for anti-inflammatory and antidepressant activity. *Curr. Drug Targets* 14 1322–1328. 10.2174/13894501113146660214 24020976

[B39] Hertz L. Song D. Li B. Du T. Xu J. Gu L. (2014). Signal transduction in astrocytes during chronic or acute treatment with drugs (SSRIs, antibipolar Drugs, GABA-ergic drugs, and benzodiazepines) ameliorating mood disorders. *J. Signal Transduct.* 2014:593934 10.1155/2014/593934 PMC3953578 24707399

[B40] Iwata K. Café-Mendes C. C. Schmitt A. Steiner J. Manabe T. Matsuzaki H. (2013). The human oligodendrocyte proteome. *Proteomics* 13 3548–3553. 10.1002/pmic.201300201 24167090

[B41] Jayasundar R. Raghunathan P. (1997). Evidence for left-right asymmetries in the proton MRS of brain in normal volunteers. *Magn. Reson. Imaging* 15 223–234. 10.1016/S0730-725X(96)00342-6 9106150

[B42] Kalkman H. O. (2011). Circumstantial evidence for a role of glutamine-synthetase in suicide. *Med. Hypotheses* 76 905–907. 10.1016/j.mehy.2011.03.005 21435795

[B43] Kato T. A. Watabe M. Kanba S. (2013). Neuron-glia interaction as a possible glue to translate the mind-brain gap: a novel multi-dimensional approach toward psychology and psychiatry. *Front. Psychiatry* 4:139 10.3389/fpsyt.2013.00139 PMC3804762 24155727

[B44] Katsel P. Byne W. Roussos P. Tan W. Siever L. Haroutunian V. (2011). Astrocyte and glutamate markers in the superficial, deep and white matter layers of the anterior cingulate gyrus in schizophrenia. *Neuropsychopharmacology* 36 1171–1177. 10.1038/npp.2010.252 21270770 PMC3077461

[B45] Kim S. Choi K. H. Baykiz A. F. Gershenfeld H. K. (2007). Suicide candidate genes associated with bipolar disorder and schizophrenia: an exploratory gene expression profiling analysis of post-mortem prefrontal cortex. *BMC Genomics* 8:413 10.1186/1471-2164-8-413 PMC2211497 17997842

[B46] Liu Z. Song D. Yan E. Verkhratsky A. Peng L. (2015). Chronic treatment with anti-bipolar drugs suppresses glutamate release from astroglial cultures. *Amino Acids* 47 1045–1051. 10.1007/s00726-015-1936-y 25676933

[B47] Luykx J. J. Laban K. G. van den Heuvel M. P. Boks M. P. Mandl R. C. Kahn R. S. (2012). Region and state specific glutamate downregulation in major depressive disorder: a meta-analysis of (1)H-MRS findings. *Neurosci. Biobehav. Rev.* 36 198–205. 10.1016/j.neubiorev.2011.05.014 21672551

[B48] Mai J. K. Paxinos G. Assheuer J. K. (2003). *Atlas of the Human Brain.* Amsterdam: Elsevier Academic Press.

[B49] Malchow B. Strocka S. Frank F. Bernstein H. G. Steiner J. Schneider-Axmann T. (2014). Stereological investigation of the posterior hippocampus in affective disorders. *J. Neural Transm.* 10.1007/s00702-014-1316-x [Epub ahead of print]. 25307869

[B50] Maletic V. Raison C. (2014). Integrated neurobiology of bipolar disorder. *Front. Psychiatry* 5:98 10.3389/fpsyt.2014.00098 PMC4142322 25202283

[B51] Manji H. K. Drevets W. C. Charney D. S. (2001). The cellular neurobiology of depression. *Nat. Med.* 7 541–547. 10.1038/87865 11329053

[B52] Martins-de-Souza D. Schmitt A. Röder R. Lebar M. Schneider-Axmann T. Falkai P. (2010). Sex-specific proteome differences in the anterior cingulate cortex of schizophrenia. *J. Psychiatr. Res.* 44 989–991. 10.1016/j.jpsychires.2010.03.003 20381070

[B53] Miguel-Hidalgo J. J. Waltzer R. Whittom A. A. Austin M. C. Rajkowska G. Stockmeier C. A. (2010). Glial and glutamatergic markers in depression, alcoholism, and their comorbidity. *J. Affect. Disord.* 127 230–240. 10.1016/j.jad.2010.06.003 20580095 PMC2975814

[B54] Mitterauer B. J. (2015). Balancing and imbalancing effects of astrocytic receptors in tripartite synapses. Common pathophysiological model of mental disorders and epilepsy. *Med. Hypotheses* 84 315–320. 10.1016/j.mehy.2015.01.025 25655220

[B55] Mosebach J. Keilhoff G. Gos T. Schiltz K. Schoeneck L. Dobrowolny H. (2013). Increased nuclear Olig1-expression in the pregenual anterior cingulate white matter of patients with major depression: a regenerative attempt to compensate oligodendrocyte loss? *J. Psychiatr. Res.* 47 1069–1079. 10.1016/j.jpsychires.2013.03.018 23615187

[B56] Öngür D. Drevets W. C. Price J. L. (1998). Glial reduction in the subgenual prefrontal cortex in mood disorders. *Proc. Natl. Acad. Sci. U.S.A.* 95 13290–13295. 10.1073/pnas.95.22.13290 9789081 PMC23786

[B57] Pehrson A. L. Sanchez C. (2015). Altered γ-aminobutyric acid neurotransmission in major depressive disorder: a critical review of the supporting evidence and the influence of serotonergic antidepressants. *Drug Des. Devel. Ther.* 9 603–624. 10.2147/DDDT.S62912 PMC4307650 25653499

[B58] Pfleiderer B. Michael N. Erfurth A. Ohrmann P. Hohmann U. Wolgast M. (2003). Effective electroconvulsive therapy reverses glutamate/glutamine deficit in the left anterior cingulum of unipolar depressed patients. *Psychiatry Res.* 122 185–192. 10.1016/S0925-4927(03)00003-9 12694892

[B59] Rajkowska G. (2000). Dysfunction in neural circuits involved in the pathophysiology of mood disorders: postmortem studies in mood disorders indicate altered numbers of neurons and glial cells. *Biol. Psychiatry* 48 766–777. 10.1016/S0006-3223(00)00950-1 11063973

[B60] Rajkowska G. (2002). Cell pathology in mood disorders. *Semin. Clin. Neuropsychiatry* 7 281–292. 10.1053/scnp.2002.35228 12382210

[B61] Rajkowska G. Miguel-Hidalgo J. J. (2007). Gliogenesis and glial pathology in depression. *CNS Neurol. Disord. Drug Targets* 6 219–233. 10.2174/187152707780619326 17511618 PMC2918806

[B62] Rajkowska G. Stockmeier C. A. (2013). Astrocyte pathology in major depressive disorder: insights from human postmortem brain tissue. *Curr. Drug Targets* 14 1225–1236. 10.2174/13894501113149990156 23469922 PMC3799810

[B63] Rowland L. M. Kontson K. West J. Edden R. A. Zhu H. Wijtenburg S. A. (2013). In vivo measurements of glutamate, GABA, and NAAG in schizophrenia. *Schizophr. Bull.* 39 1096–1104. 10.1093/schbul/sbs092 23081992 PMC3756774

[B64] Sahlender D. A. Savtchouk I. Volterra A. (2014). What do we know about gliotransmitter release from astrocytes? *Philos. Trans. R. Soc. Lond. B Biol. Sci.* 369:20130592 10.1098/rstb.2013.0592 PMC4173278 25225086

[B65] Salvadore G. van der Veen J. W. Zhang Y. Marenco S. Machado-Vieira R. Baumann J. (2012). An investigation of amino-acid neurotransmitters as potential predictors of clinical improvement to ketamine in depression. *Int. J. Neuropsychopharmacol.* 15 1063–1072. 10.1017/S1461145711001593 22040773 PMC3342437

[B66] Savitz J. B. Price J. L. Drevets W. C. (2014). Neuropathological and neuromorphometric abnormalities in bipolar disorder: view from the medial prefrontal cortical network. *Neurosci. Biobehav. Rev.* 42 132–147. 10.1016/j.neubiorev.2014.02.008 24603026

[B67] Schitine C. Nogaroli L. Costa M. R. Hedin-Pereira C. (2015). Astrocyte heterogeneity in the brain: from development to disease. *Front. Cell. Neurosci.* 9:76 10.3389/fncel.2015.00076 PMC4367182 25852472

[B68] Schroeter M. L. Steiner J. Schönknecht P. Mueller K. (2014). Further evidence for a role of S100B in mood disorders: a human gene expression mega-analysis. *J. Psychiatr. Res.* 53 84–86. 10.1016/j.jpsychires.2014.02.021 24629352

[B69] Sequeira A. Mamdani F. Ernst C. Vawter M. P. Bunney W. E. Lebel V. (2009). Global brain gene expression analysis links glutamatergic and GABAergic alterations to suicide and major depression. *PLoS ONE* 4:e6585 10.1371/journal.pone.0006585 PMC2719799 19668376

[B70] Shende V. H. McArthur S. Gillies G. E. Opacka-Juffry J. (2015). Astroglial plasticity is implicated in hippocampal remodelling in adult rats exposed to antenatal dexamethasone. *Neural Plast.* (in press) 10.1155/2015/694347 PMC4539493 26345609

[B71] Si X. Miguel-Hidalgo J. J. O’Dwyer G. Stockmeier C. A. Rajkowska G. (2004). Age-dependent reductions in the level of glial fibrillary acidic protein in the prefrontal cortex in major depression. *Neuropsychopharmacology* 29 2088–2096. 10.1038/sj.npp.1300525 15238995 PMC3146059

[B72] Skolnick P. Popik P. Trullas R. (2009). Glutamate-based antidepressants: 20 years on. *Trends Pharmacol. Sci.* 30 563–569. 10.1016/j.tips.2009.09.002 19837463

[B73] Sofroniew M. V. Vinters H. V. (2010). Astrocytes: biology and pathology. *Acta Neuropathol.* 119 7–35. 10.1007/s00401-009-0619-8 20012068 PMC2799634

[B74] Steiner J. Bogerts B. Sarnyai Z. Walter M. Gos T. Bernstein H. G. (2012). Bridging the gap between the immune and glutamate hypotheses of schizophrenia and major depression: potential role of glial NMDA receptor modulators and impaired blood-brain barrier integrity. *World J. Biol. Psychiatry* 13 482–492. 10.3109/15622975.2011.583941 21707463

[B75] Takasaki C. Yamasaki M. Uchigashima M. Konno K. Yanagawa Y. Watanabe M. (2010). Cytochemical and cytological properties of perineuronal oligodendrocytes in the mouse cortex. *Eur. J. Neurosci.* 32 1326–1336. 10.1111/j.1460-9568.2010.07377.x 20846325

[B76] Teschemacher A. G. Gourine A. V. Kasparov S. (2015). A role for astrocytes in sensing the brain microenvironment and neuro-metabolic integration. *Neurochem. Res.* 10.1007/s11064-015-1562-9 [Epub ahead of print] 25837670

[B77] Thompson S. M. Kallarackal A. J. Kvarta M. D. Van Dyke A. M. LeGates T. A. Cai X. (2015). An excitatory synapse hypothesis of depression. *Trends Neurosci.* 38 279–294. 10.1016/j.tins.2015.03.003 25887240 PMC4417609

[B78] Toro C. T. Hallak J. E. Dunham J. S. Deakin J. F. (2006). Glial fibrillary acidic protein and glutamine synthetase in subregions of prefrontal cortex in schizophrenia and mood disorder. *Neurosci. Lett.* 404 276–281. 10.1016/j.neulet.2006.05.067 16842914

[B79] Uranova N. A. Vostrikov V. M. Orlovskaya D. D. Rachmanova V. I. (2004). Oligodendroglial density in the prefrontal cortex in schizophrenia and mood disorders: a study from the Stanley Neuropathology Consortium. *Schizophr. Res.* 67 269–275. 10.1016/S0920-9964(03)00181-6 14984887

[B80] Van Horn M. R. Sild M. Ruthazer E. S. (2013). D-serine as a gliotransmitter and its roles in brain development and disease. *Front. Cell. Neurosci.* 7:39 10.3389/fncel.2013.00039 PMC3632749 23630460

[B81] Verkhratsky A. Rodríguez J. J. Steardo L. (2014). Astrogliopathology: a central element of neuropsychiatric diseases? *Neuroscientist* 20 576–588. 10.1177/1073858413510208 24301046

[B82] Vostrikov V. M. Uranova N. A. Orlovskaya D. D. (2007). Deficit of perineuronal oligodendrocytes in the prefrontal cortex in schizophrenia and mood disorders. *Schizophr. Res.* 94 273–280. 10.1016/j.schres.2007.04.014 17566708

[B83] Walter M. Henning A. Grimm S. Schulte R. F. Beck J. Dydak U. (2009). The relationship between aberrant neuronal activation in the pregenual anterior cingulate, altered glutamatergic metabolism, and anhedonia in major depression. *Arch. Gen. Psychiatry* 66 478–486. 10.1001/archgenpsychiatry.2009.39 19414707

[B84] Xu J. Dydak U. Harezlak J. Nixon J. Dzemidzic M. Gunn A. D. (2013). Neurochemical abnormalities in unmedicated bipolar depression and mania: a 2D 1H MRS investigation. *Psychiatry Res.* 30 235–241. 10.1016/j.pscychresns.2013.02.008 23810639 PMC3729606

[B85] Yüksel C. Öngür D. (2010). Magnetic resonance spectroscopy studies of glutamate-related abnormalities in mood disorders. *Biol. Psychiatry* 68 785–794. 10.1016/j.biopsych.2010.06.016 20728076 PMC2955841

